# Interdisciplinary Development and Fine-Tuning of CARDIO, a Large Language Model for Cardiovascular Health Education in HIV Care: Tutorial

**DOI:** 10.2196/77053

**Published:** 2025-09-12

**Authors:** Ryan Rullo, Ali Maatouk, Tinglin Huang, Jialin Chen, Weikang Qiu, Giselle O'Connor, Julie Womack, Tatiana Sadak, Christine Rodriguez, Pedro Carneiro, Tania de Jesus Espinosa, Ami Marshall, Rex Ying, S Raquel Ramos

**Affiliations:** 1 School of Nursing Yale University Orange, CT United States; 2 Department of Computer Science School of Engineering and Applied Science Yale University New Haven, CT United States; 3 VA Connecticut Healthcare System West Haven, CT United States; 4 Department of Social and Behavioral Sciences School of Public Health Yale University New Haven, CT United States

**Keywords:** artificial intelligence, HIV, cardiovascular health, health promotion, large language models, patient education, discharge education, interdisciplinary research, cardiovascular risk, nursing, computer science

## Abstract

**Background:**

The integration of artificial intelligence in health care presents a significant opportunity to revolutionize patient care. In the United States, an estimated 129 million people have at least 1 chronic illness, with 42% having 2 or more. Despite being largely preventable, the prevalence of chronic illness is expected to rise and impose significant economic burdens and financial toxicity on health care consumers.

**Objective:**

We leveraged an interdisciplinary team encompassing nursing, public health, and computer science to optimize health through prevention education for cardiovascular and metabolic comorbidities in persons living with HIV. In this tutorial, we describe the iterative, data-based development and evaluation of an intersectionality-informed large language model designed to support patient teaching in this population.

**Methods:**

First, we curated data by scraping publicly available, authoritative, evidence-based sources to capture a comprehensive dataset, supplemented by publicly available HIV forum content. Second, we benchmarked candidate large language models and generated a fine-tuning dataset using GPT-4 through multiturn question and answer conversations, using standardized metrics to assess baseline model performance. Third, we iteratively refined the selected model via low-rank adaptation and reinforcement learning, integrating quantitative metrics with qualitative expert evaluations.

**Results:**

Pre-existing large language models (LLMs) demonstrated poor n-gram agreement, dissonance from model answers (accuracy 4.16, readability 4.63, and professionalism 4.58), and difficult readability (Kincaid 8.54 and Jargon 4.44). After prompt adjustments and fine-tuning, preliminary results demonstrate the potential of a customized Llama-based LLM to provide personalized, culturally salient patient education.

**Conclusions:**

We present a data-based, step-by-step tutorial for interdisciplinary development of CARDIO, a specialized LLM, for cardiovascular health education in HIV care. Through comprehensive data curation and scraping, systematic benchmarking, and a dual-stage fine-tuning pipeline, CARDIO’s performance improved markedly (accuracy 5.0, readability 4.98, professionalism 4.98, Kincaid 7.17, and Jargon 2.92). Although patient pilot testing remains forthcoming, our results demonstrate that targeted data curation, rigorous benchmarking, and iterative fine-tuning have provided a robust evaluation of the model’s potential. By building an LLM tailored to cardiovascular health promotion and patient education, this work lays the foundation for innovative artificial intelligence–driven strategies to manage comorbid conditions in people living with HIV.

## Introduction

### Background

In recent years, there has been a substantive advancement in the use of artificial intelligence (AI) in health care settings. Technological improvements in both natural language processing and machine learning (ML) have positioned AI to revolutionize patient care [1-4]. In nursing, AI can include decision-making tools, educational study materials, patient chatbots, electronic health record notifications, and more [2,5,6]. While AI could enhance nursing practice, it is not without risks. Ethical concerns regarding AI in health care highlight risks in the development, reliability, integration, and confidentiality of these models [3-8]. For 23 consecutive years, nursing has been ranked as the most ethical and honest profession in the United States [9]. Due to this, nursing must be involved in researching, developing, and implementing cutting-edge technologies in patient care, such as AI, to enhance care competence and ensure safety for all persons [10,11].

In the United States, 129 million people are estimated to have 1 chronic illness, and 42% of those individuals have 2 or more chronic conditions [12]. Despite many of these conditions being preventable, the prevalence of chronic illness is expected to increase [13]. The economic impact on health care and the financial toxicity placed on patients are significant [14]. The most prevalent chronic diseases include cardiovascular disease (CVD) and diabetes, which are estimated to cost the United States over 1 trillion dollars annually [13]. While there is movement in the health care field toward upstream preventative measures to mitigate the prevalence and cost of chronic disease, these efforts are limited by structural, social, and environmental factors. Moreover, findings from a longitudinal, multisite, cohort study of 3972 persons living with HIV suggested that a visit to a cardiology clinic was not linked to improvements in CVD risk factors or a decreased risk of major adverse cardiovascular events [15]

Social determinants of health are nonmedical factors (housing, insurance, education, etc.) that contribute to well-being and drive health outcomes [16]. However, sociodemographic factors, such as education, race, health care access, living situation, and income, have been connected to chronic disease prevalence in many populations [12,16-18]. Staggering differences exist when considering that persons from diverse backgrounds have twice the likelihood of death from CVD [14,19] and stroke [20] than their counterparts [13]. This is true for groups with multifaceted and interconnected identities [21,22] as well as older adolescents entering adulthood [23]. These differences may be attributed to minority stress (chronic stress from bias and stigma), varied health care access, and heightened cardiovascular risk [14,24-26].

Persons with HIV are at an increased risk of CVD, which remains a leading cause of death worldwide, with an estimated 32% cause rate in 2020 [13,20]. Risk factors for CVD, such as hypertension and diabetes [20], are also increasing in prevalence, with diabetes now being the eighth leading cause of death [13], particularly in diverse groups [27]. Persons with HIV are at an increased risk of CVD due to chronic inflammation from HIV infection, hyperlipidemia-causing medications, and lifestyle factors [28]. The potential to mitigate these disparities through technologies that have been gaining significant uptake in patient care, such as AI, is vital for advancing health optimization.

### Current State of AI for HIV and Cardiovascular Care

Cardiometabolic health is the body’s ability to manage risk factors, such as blood pressure, blood sugar, cholesterol, and weight to reduce the risk of heart disease, stroke, and diabetes [21]. Despite an estimated 1.2 million people living with HIV in the United States [27], there is a limited presence in health care of AI-developed patient education tools for cardiovascular health promotion and behavioral disease prevention. The use of AI in health care has predominantly used ML and deep learning to develop and train prediction models, screening tools [29,30], and patient education adjuncts to help decrease new infections and manage current illnesses [2,4,31,32]. Using deep neural networks to develop ML models can help predict CVD risk in people with HIV [33], identify candidates for pre-exposure prophylaxis [34], and monitor treatment adherence [7]. Furthermore, AI can be used to guide treatment plans, provide personalized reminders, and serve as chatbots for basic questions [7]. Chatbots specifically have been used successfully in sexual health because of their ease of use, availability, and accessibility. However, there are concerns regarding understandability and accuracy [35]. These same issues are found in chatbots for cardiovascular care prevention [36]. ML-based chatbots may exhibit limited conversational understanding, provide inflexible topic options, and present biases and safety risks [37,38]. However, there is promise in developing personalized large language models (LLMs) to mitigate these limitations in areas, such as prostate cancer [39] and ophthalmology [39,40]. While these methods require further development, one of the potentially largest opportunities for AI in health care is in improving patient education using an interdisciplinary lens.

In order to gain a better understanding of AI, it’s important to first comprehend the ways AI is described. Common terms in the understanding and application of AI are defined in Table 1.

**Table 1:** Common terms and definitions related to artificial intelligence (AI).

Term	Definition
AI	A computer’s ability to do tasks that normally need human thinking, such as recognizing patterns, solving problems, or making decisions.
Deep learning	A subset of machine learning that uses neural networks, often multilayered, to analyze complex data and learn patterns. It is inspired by the structure of our human brains.
Large language model	A type of AI model trained on massive amounts of text data to understand, generate, and respond similarly to how humans talk or write.
Machine learning	A subset of AI where machines learn from data and improve their performance over time without explicit programming.
Natural language processing	The ability of AI to understand, interpret, and respond to human language—spoken or written.
Neural networks	A set of algorithms designed to work like the human brain, helping computers to learn from data.
Scraping	Extraction of information from websites. It is often used to gather large amounts of data to train AI models.
Tokens	Small chunks of text (words or parts of words) that AI uses to process and understand language.

### Current State of AI for Patient Education in the Health Care Setting

Patient readmission continues to be a substantial cost for hospitals and the health care industry [41,42]. While discharge education could reduce readmission rates, there are substantial barriers to successful discharge education, such as limited learning assessments, standardized education forms, and limited nursing time [42,43]. Patients are estimated to spend around 5 minutes with providers and 20 minutes with nursing staff on the day of discharge [44]. It is estimated that fewer than 60% of patients understand their diagnosis after discharge, and less than 44% can recall appointment details [45]. Given the limited understanding of discharge instructions and the use of advanced terms by providers to describe illness [45], it is understandable why patients may be uncertain of how to appropriately integrate health teaching upon leaving the hospital setting. Studies suggest that there may be substantial value in using AI in postdischarge education and monitoring [46-50]. AI-generated discharge summaries are promising for patient understanding and personalization when compared to standardized templates currently used by clinicians [46,47]. However, any AI model is only as good as the data on which it is trained [7].

### Bias and Stereotypes in AI

LLMs are interrelated with AI, ML, and data science. The process of creating an LLM requires obtaining high-quality data, fine-tuning pre-existing models, and evaluating those models against specific metrics to ensure accuracy, usability, and performance [5]. Despite the large potential of LLMs to revolutionize many sectors, including health care, there are substantial risks to their implementation [48-51]. If the materials on which the LLM is trained are biased, the outcomes of the LLM may perpetuate these biases and limit personalization [5,49,50]. To mitigate these harms, it is important to use diverse datasets and bias-mitigation prompts and obtain user feedback [5,51]. In addition, testing for data refusals, offensive autocompletes, and toxic responses can help determine which LLM to fine-tune, with Llama and Mistral potentially performing the best [49]. Additional ethical concerns with LLMs include false information (ie, “hallucinations”), security and privacy risks, natural resource depletion, transparency in algorithm development and function (ie, “black box”), and long-term cultural effects [5,51,52]. These risks are multiplicative when considering intersectional identities and perpetuating minority stress in already vulnerable populations.

This project uses an interdisciplinary team of nurses, public health professionals, and computer scientists to address how intersecting social and structural determinants shape CVD in populations with HIV. The purpose of this tutorial is to describe the iterative steps of developing a novel, intersectionality-based LLM to promote cardiovascular health among persons with HIV. To date, the authors were unable to identify other studies that integrate an intersectional approach into LLM development for enhancing cardiovascular health in persons with HIV.

## Methods

### Guiding Frameworks for Model Development: Intersectionality and Minority Stress

First introduced by Crenshaw [53,54], intersectionality describes how overlapping marginalized identities can compound experiences of discrimination and oppression. Experiences of discrimination and social stigma create a hostile environment for marginalized groups, which can influence health outcomes, as described in minority stress theory [55,56]. While this theory has been readily used in research, it does not necessarily apply to intersectional identities, nor does it adapt to cultural changes over time [57]. Therefore, both the minority stress theory and intersectionality serve as a basis for the model training and resource selection in our LLM, ensuring that all persons can benefit from its use.

### Health Promotion and Behavioral Change: American Heart Association Life’s Essential 8 Framework

The American Heart Association (AHA) is a leading authority for its research, guidelines, and educational resources related to cardiovascular health and disease prevention. The AHA Life’s Essential 8 was selected to guide this work, given that it is grounded in the evidence, clinically relevant, and uses a multidimensional lens to develop cardiovascular guidelines and resources for both health care professionals and the public [21-23]. The American Heart Association's Life's Essential 8 framework offers a comprehensive approach to promoting cardiovascular health by encompassing 8 primary domains, including diet, physical activity, tobacco use, sleep, weight, cholesterol, diabetes, and hypertension [58]. Each domain is integral to reducing the risk of chronic illness, such as heart disease and stroke. This work maintains the focus on cardiovascular health promotion by using these domains to structure the data collection process and overall project objectives.

### Step 1: Team Development

**Figure 1:**
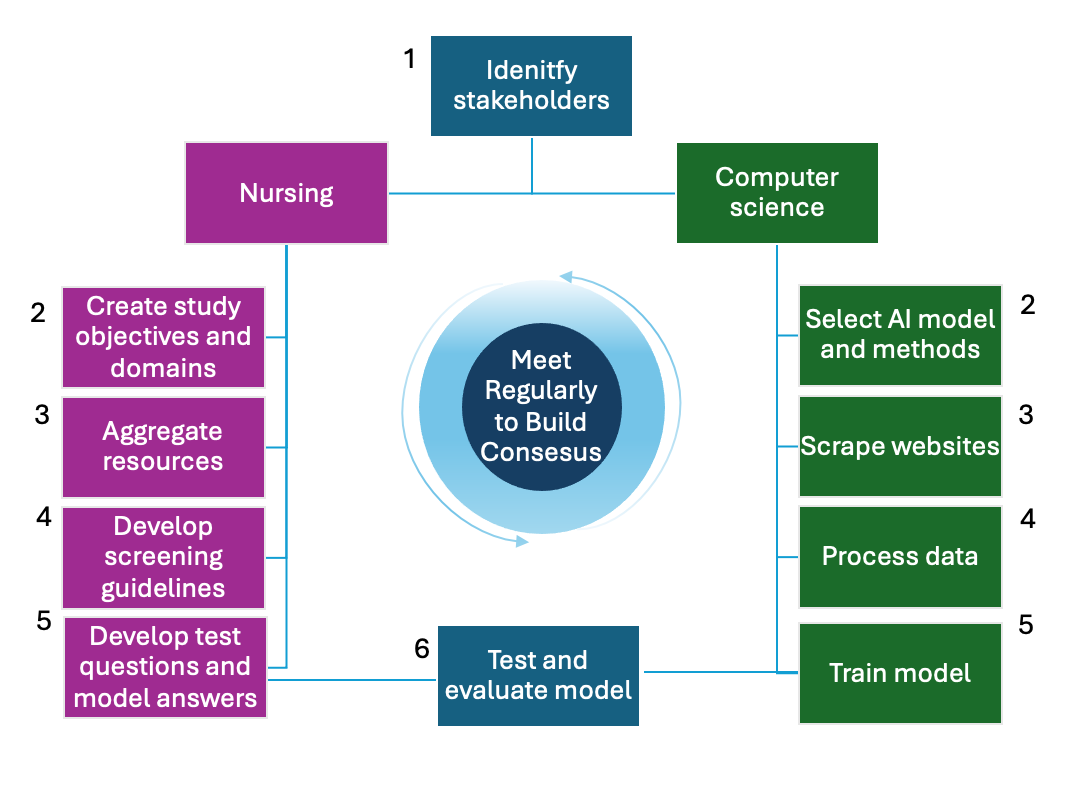
Large language model development process diagram.

To attain our goal for LLM development, interdisciplinary collaboration was crucial throughout each step of the process. Interdisciplinary collaboration differs from multidisciplinary or transdisciplinary approaches as it integrates diverse knowledge bases to achieve a shared goal [59]. The team development process is described in Figure 1, and each step required multiple substeps for completion. The first was to develop our team, which consisted of experts in nursing, public health, and computer science (CS; Table 2). The team had members representing various identities as well as research backgrounds in health disparities research and behavioral health interventions.

To begin, the health care professional (HCP) team collaborated to create study objectives and aggregate resources across the domains. After reviewing the resources, the CS team selected the best methods for scraping and benchmarking. The HCP team periodically reviewed the scraped websites to ensure accuracy. While the CS team processed data and began training the selected model, the HCP team created safety guidelines and sample question and answer pairs. While both teams had their respective activities, our weekly meetings proved to be the most fruitful component of this process. To truly collaborate, a shared language had to be developed between the 2 disciplines, as many terms of art were not mutually understandable. By sharing goals and expectations and defining key metrics, we were able to develop a successful, productive team dynamic. The back-and-forth communication between disciplines was key in training and adapting the model. Expert external consultants were added to the team to test the model and provide insight prior to piloting for end-user feedback.

**Table 2:** Team members and stakeholders.

Role	Degree	Research background
Nursing lead	PhD^a^, MBA^b^, MSN^c^	Behavioral interventions, CVD^d^, HIV, consumer health informatics, and populations with multifaceted identities
CS^e^ Lead	PhD	Graph learning and foundation models, AI^f^: LLM^g^, ML^h^
Project Manager	MPH^i^	Public health, digital health, HIV, and lifestyle interventions
CS Post Doc^j^	PhD	Information theory, optimization, and foundational models
CS GRA^k^ 1	MSc^l^	AI for science and LLM development
CS GRA 2	MSc	AI for science and ML development
CS GRA 3	MSc	Foundation models, time series analysis, and trustworthy AI
Nursing GRA^m^ 1	MSN	Health care clinician, HIV, and cancer prevention, cancer care
Nursing GRA 2	MSN	Health care clinician, populations with multifaceted identities, nursing education, and virtual reality education
Nursing Post Doc^n^	PhD, MPH	Public health, populations with multifaceted identities, implementation science, and big data, patient-reported outcomes
Expert Consultant	PhD	Psychiatric advanced practice RN^o^, mental health, and aging
Expert Consultant	EdD^p^	Medical director, advanced practice nurse, clinical care, and education
Expert Consultant	DNP^q^	Virtual reality, augmented reality, AI, clinical simulation, advanced practice clinician, and populations with multifaceted identities
Expert Consultant	PhD	Informatics, ML, big data, aging, and HIV

^a^PhD: Doctor of Philosophy.

^b^MBA: Master of Business Administration.

^c^MSN: Master of Science in Nursing.

^d^CVD: cardiovascular disease.

^e^CS: computer science.

^f^AI: artificial intelligence.

^g^LLM: large language model.

^h^ML: machine learning.

^i^MPH: Master of Public Health.

^j^CS Post Doc: Computer Science Postdoctoral Researcher.

^k^CS GRA: Computer Science Graduate Research Assistant.

^l^MSc: Master of Science.

^m^GRA: Graduate Research Assistant.

^n^Post Doc: Postdoctoral Researcher.

^o^RN: Registered Nurse.

^p^EdD: Doctor of Education.

^q^DNP: Doctor of Nursing Practice.

### Step 2: Data Curation

To develop the dataset, we began by creating objectives for the project and dividing these into primary domains, guided by the AHA Essential 8 (Table 3). Then, we collected publicly available, evidence-based health information on CVD prevention, HIV care, and general health knowledge. We refined our search criteria to include internet-based, patient- and provider-facing sites, putting emphasis on validated sources of literature, such as research papers and governmental websites. By focusing on authoritative websites, such as AHA’s Essential 8, health screening guidelines, official health websites (eg, Centers for Disease Control and Prevention [CDC] and World Health Organization [WHO], and current US legislation, we obtained a robust catalog of resources (Table S1 in Multimedia Appendix 1). The entirety of PubMed was included for current scientific research. Certain resources were specifically added to mitigate bias and promote patient safety. These included harm-reduction strategies, therapeutic communication techniques, mental health screenings, and sexual and gender inclusive data. To mitigate copyright concerns, all data sources used were publicly available and either patient or provider-facing. To integrate intersectional patient experiences, we also sampled publicly available HIV forums to formulate baseline knowledge of patient-expressed needs, concerns, and questions. Further, we gathered targeted materials to address our cardiovascular subobjective regarding populations' experiences with health disparities so that it is useful for every individual. This included current legislature and provider directories to build in political context and safety features.

Data scraping was far more complex than simply downloading a single HTML page. In most cases, it required scraping entire websites, as valuable information was dispersed across multiple pages. In addition, some websites hosted important content in PDF format, which was also scraped. Due to restrictions on automatic scraping by some websites, several of these PDFs had to be extracted manually. Traditional processors (pdfminer, pypdf, etc), HTML parsers (eg, Beautiful Soup), Adobe Toolbox, and human refinement were combined to accurately scrape data sites [60-62]. Once this collection of PDFs and text files was assembled, the data was standardized using Microsoft’s Markitdown tool to convert PDFs into plain text [62]. This tool was chosen following an ablation study comparing various alternatives, including PyPDF [61] and PyMuPDF [60], where Markitdown demonstrated the best performance, as validated by both teams. One major challenge during this conversion process, however, was interpreting tables due to wrong formatting. In fact, without proper formatting, table data could be misleading. As a result, we manually edited these tables to align them with their original formatting or removed them altogether if the information was redundant in the document

**Table 3:** Study domains and objectives.

Domain	Main objective	Subobjective
Sleep	Improve understanding and management of sleep as a risk factor for HIV comorbidities.	Provide evidence-based information regarding sleep hygiene. Provide information about the relationship between sleep and health.
Exercise	Promote physical activity as a preventive measure to improve overall health.	Provide evidence-based information about the effects of exercise on the body. Educate about the integration of physical activity in daily life.
Smoking	Reduce tobacco and nicotine use as a shared risk factor for CVD^a^ and cancer.	Provide evidence-based information regarding harmful effects of tobacco and nicotine use. Provide helpful instructions on how to quit smoking.
Weight	Address the role of weight management in mitigating comorbidities among people with HIV.	Promote healthy weight practices to improve health.
Diet	Improve dietary practices to reduce comorbidities in persons with HIV.	Provide evidence-based information regarding healthy food choices, portion sizes, nutrition labels, and dietary strategies.
Hypertension	Increase awareness and prevention of hypertension as a leading HIV comorbidity.	Provide evidence-based information regarding BP^b^ levels, risk factors, complications, and management strategies.
Mental health	Address mental health as a critical factor in managing HIV and related comorbidities.	Provide evidence-based information regarding mental health. Provide tools and resources relating to mental health.
Cardiovascular general	Reduce the burden of CVD and CVD risk among persons with HIV.	Provide evidence-based information regarding common cardiac conditions (MI^c^, HF^d^, AF^e^, and stroke) Consider SDOH^f^, ethnic and racial disparities, and intersectionality.
T2DM^g^	Prevent and manage type 2 diabetes among people with HIV.	Provide evidence-based information regarding glucose levels, risk factors, complications, and management strategies.
Cholesterol	Manage cholesterol levels to reduce cardiovascular risk in people with HIV.	Provide evidence-based information regarding cholesterol levels, risk factors, complications, and management strategies
HIV care	Ensure comprehensive HIV care that addresses physical and mental health comorbidities.	Provide evidence-based information and resources relating to HIV care across the continuum.
Screenings/ prevention	Promote early detection and prevention of HIV-related comorbidities.	Provide evidence-based information and resources related to PrEP^h^ and cancer prevention.
Sexual and gender inclusive data^i^ general	Ensure that differences in individual characteristics are considered in policy and clinical practice.	Provide individuals with personalized information regarding health care legislation in their state. Provide targeted support and information on providers in their state.

^a^CVD: cardiovascular disease.

^b^BP: blood pressure.

^c^MI: myocardial infarction.

^d^HF: heart failure.

^e^AF: atrial fibrillation.

^f^SDOH: social determinants of health.

^g^T2DM: type 2 diabetes mellitus.

^h^PrEP: pre-exposure prophylaxis.

### Step 3: Benchmarking

To develop the model, we compiled current LLMs available for fine-tuning. The selection process would be based on benchmarked scores for information accuracy and model performance [63,64]. The LLMs selected for benchmarking are listed and briefly defined by our CS team in Table 4. With the necessary resources compiled, we initiated the benchmarking process. For this effort, we focused on models of reasonable size that support local inference, such as BioMistral [65], Qwen2.5 [66], and Meta’s Llama 3.1 [67]. This initial comparison helped us identify models demonstrating strong baseline performance. Then, the CS team curated a specialized fine-tuning dataset to further enhance the model’s behavior. This dataset was generated using GPT-4o [68], which produced multiturn Q&A conversations based on the content gathered from the websites. Instructions were given to GPT-4o to create this dataset with an accessible reading level while avoiding complex medical terminology. Using this dataset, we fine-tuned the selected model via the low-rank adaptation of the LLM fine-tuning method to improve its alignment and overall performance [69]. Finally, we applied reinforcement learning using Group Relative Policy Optimization, with reward signals that incentivize the model to generate responses at or around a fifth-grade Flesch-Kincaid reading level and to avoid the use of technical medical language [70].

**Table 4:** Large language models selected for benchmarking.

Model	Description
Vicuna-7B	An open-source large language model with 7 billion parameters, trained to act like a helpful assistant.
BioMistral-7B	A medical-focused large language model with 7 billion parameters. It is trained on health-related texts to better understand medical language.
Qwen2.5-7B	A general-purpose language model with 7 billion parameters, designed for everyday conversation and reasoning tasks.
Qwen 2.4-14B	A larger version of Qwen2, with 14 billion parameters, offering better performance on more complex language tasks.
Llama3.1-8B	A newer version of Meta’s large language model (Llama), with 8 billion parameters, designed to be faster and more accurate.
Deepseek -R1-Llama8B	A hybrid model combining Deepseek’s training approach with Llama’s architecture. It has 8 billion parameters and is used for general-purpose tasks.
Deepseek-R1-Qwen32B	A hybrid model combining Deepseek’s training approach with Llama’s architecture. It has 8 billion parameters and is used for general-purpose tasks.

### Iterative Refinements

There were multiple iterations and methods of measurement for testing the LLM. Overall, we wanted to ensure informational accuracy, interpretability, and safety. To reach these goals, we created a rubric to evaluate model answers (Table S1 in Multimedia Appendix 2) and generated expert-developed sample answers (Table S1 in Multimedia Appendix 3). In addition, we integrated mental health algorithms to prompt screenings and emergency services connections for concerning language on substance abuse, alcohol abuse, anxiety, depression, and suicidal thoughts (Figures S1 and S2 in Multimedia Appendix 4). Furthermore, we based the interpretability of the model on Flesch-Kincaid reading scores and medical jargon usage, with a goal of third- to fifth-grade reading level [71,72]. To assess the LLM, we developed a curated set of expert-generated question and answer (QA) pairs. Half were created by Expert 1 (RR) and half by Expert 2 (TJE), with each expert reviewing and refining the other’s contributions to ensure interrater reliability (Table S1 in Multimedia Appendix 5). This high-quality dataset served as part of the initial training input for the model. Following the initial training phase, we prompted the model to independently generate new QA pairs. These model-generated responses were then evaluated against expert-authored answers. Both experts independently reviewed the model’s output for accuracy, clarity, and alignment with clinical best practices. Model refinement was iterative. Each time the model was updated, a new batch of sample QAs was reviewed by the experts. This process is ongoing until there is a consistent consensus between the model-generated answers and the expert expectations, ensuring that the LLM demonstrates reliable, safe, and clinically appropriate reasoning across a range of question types.

### Ethical Considerations

Institutional Review Board approval was obtained from Yale University on January 25, 2025 (2000038443).

## Results

### Resource Scraping

We had a total of 868 PDF files, contributing to 7 million tokens and 130 QA pairs. Of note, some of the data sources we used have been removed from publication due to changes in federal research priorities, which did not impact this work but may impact future LLM iterations.

### Assessment of Source Materials

Initially, the readability of the resource text was poor. An average Flesch Reading Ease Score of 26.53 for the selected documents equates to the “Very Confusing” level [72]. This is likely because of the medical reliability of selected websites. The distribution of text sentiment, from -1 (negative opinion) to +1 (positive opinion), varied substantially in our resource material , likely stemming from both risk-focused and prevention-focused sources [73]. Based on these findings, we also created a professional score to identify medical jargon and advanced sentence structure, which combined the percentage of medical terms and complex sentences in the responses. Our goal was to continue fine-tuning our model to reach a third- to fifth-grade reading level while maintaining sentiment and limiting medical jargon.

### Benchmarking

The initial evaluation metrics chosen were based on n-gram precision. An n-gram is the word-for-word alignment of a text. Bilingual Evaluation Understudy (BLEU) measures how many n-grams match the generated text [74]. For example, BLEU 1 measures a single-word alignment, and BLEU 4 measures matching 4-word sequences, respectively. The Metric for Evaluation of Translation with Explicit Ordering (METEOR) is a similar metric that uses synonyms, paraphrasing, and stemmed matches rather than just word-for-word alignment [75]. The Recall-Oriented Understudy for Gisting Evaluation (ROUGE) is a recall-based overlap score between generated and reference text [76]. These metrics provide a comparison between readily available models to find optimal performance for further fine-tuning. Unfortunately, no pretrained model demonstrated substantial alignment with reference answers (Table 5). This is likely due to a disagreement between our requested response readability and the professionally written reference material. Therefore, Llama-3.1-8B was selected as the base model for its superior prompt-following capabilities, strong multilingual performance in English and Spanish, and natural conversational flow [67]. It also maintains a relatively modest parameter count, thus enabling efficient local computation and reducing environmental resource consumption [67].

**Table 5:** Benchmarked large language model performance.

Metric	Model						
	Vicuna-7b	BioMistral-7B	Qwen2.5-7B	Qwen2.5-14B	llama3.1-8B	Deepseek-R1 llama8B	Deepseek-R1 -qwen32B
bleu@1	0.1818	0.2109	0.1261	0.1401	0.1365	0.1342	0.1717
bleu@2	0.1051	0.1151	0.0638	0.0751	0.0690	0.0735	0.1036
bleu@3	0.0667	0.0687	0.0359	0.0440	0.0384	0.0441	0.0677
bleu@4	0.0453	0.0428	0.0213	0.0272	0.0228	0.0279	0.0465
meteor	0.1811	0.1051	0.1566	0.1710	0.1290	0.1586	0.1814
rouge	0.1912	0.1737	0.1338	0.1474	0.1387	0.1424	0.1917

### Expert Review and Fine Tuning

Question and answer (QA) pairs were generated by each LLM iteration and sent to health care experts to determine accuracy and interpretability. We then optimized the policy using Group Relative Policy Optimization (Figure S1 in Multimedia Appendix 6). The initial responses were substantially limited and required multiple rounds of fine-tuning before a consensus between expert and model responses was found

### Sample of QA Pairs

Initial LLM responses were long, medically complex, and sometimes provided information that should be left to health care providers. While the fine-tuning process is ongoing, the current responses were shorter and demonstrate improved readability. The examples are listed in Textbox 1.

**Question: How does sleep duration impact cardiovascular health?**

**Untrained large language model answer**

Sleep duration has a significant impact on cardiovascular health. Research has consistently shown that both short and long sleep durations can increase the risk of cardiovascular disease. Here are some ways in which sleep duration affects cardiovascular health. Short sleep duration (less than 7 hours per night): Increased blood pressure: Short sleep duration is associated with higher blood pressure, which is a major risk factor for cardiovascular disease; Inflammation: Short sleep duration can lead to chronic inflammation, which can damage blood vessels and increase the risk of cardiovascular disease; Cardiac arrhythmias: Short sleep duration has been linked to an increased risk of cardiac arrhythmias, such as atrial fibrillation; Cardiovascular mortality: Studies have shown that short sleep duration is associated with an increased risk of cardiovascular mortality, Long sleep duration (more than 9 hours per night): Long sleep duration has been linked to an increased risk of cardiovascular disease, including heart failure, coronary artery disease, and stroke; Obesity and metabolic changes: Long sleep duration can lead to weight gain and metabolic changes, which can increase the risk of cardiovascular disease; Inflammation and oxidative stress: Long sleep duration can lead to chronic inflammation and oxidative stress, which can damage blood vessels and increase the risk of cardiovascular disease; Cardiac arrhythmias: Long sleep duration has also been linked to an increased risk of cardiac arrhythmias, such as atrial fibrillation. Optimal sleep duration for cardiovascular health: The American Heart Association recommends 7-8 hours of sleep per night for optimal cardiovascular health. Consistency: Consistency is key when it comes to sleep duration. Aim to go to bed and wake up at the same time every day, including weekends. Quality sleep: Prioritize quality sleep by creating a sleep-conducive environment, avoiding caffeine and electronics before bedtime, and engaging in relaxing activities before bed. In summary, both short and long sleep durations can have negative effects on cardiovascular health. Aim for 7-8 hours of sleep per night and prioritize quality sleep to reduce the risk of cardiovascular disease.

**Fine-tuned large language model answer**

Sleep duration has a significant impact on cardiovascular health. Getting adequate sleep is crucial for maintaining a healthy heart and blood vessels. Research has shown that both too little and too much sleep can be detrimental to cardiovascular health. Sleeping less than 7 hours per night can increase the risk of high blood pressure, heart disease, and stroke. This is because sleep deprivation can lead to increased levels of stress hormones like cortisol and adrenaline, which can raise blood pressure and increase the risk of cardiovascular disease. On the other hand, sleeping more than 9 hours per night can also be problematic. Excessive sleep has been linked to an increased risk of cardiovascular disease, as well as other health issues like diabetes and obesity.

### Fine-Tuning Results

Fine-tuning (Table 6), including prompt editing, further training, and rewards, improved the accuracy, readability, and jargon use of our Llama-based model. Given the readability goals for our LLM output compared to the professional language of input materials, n-gram agreement scores (BLEU, METEOR, and ROUGE) were determined to be unsatisfactory metrics. Therefore, we developed separate metrics upon which the LLM would be evaluated. For the purposes of fine-tuning, accuracy is defined as agreement with expert-provided sample answers (1 being the lowest agreement and 5 being the highest agreement). Readability is defined as agreement with the advised patient-teaching language metrics, including syllable count, words per sentence, and sentence structure (1 being the lowest agreement and 5 being the highest agreement). Professional score is defined as the level of personalization and bias-free language (1 being the lowest agreement and 5 being the highest agreement). The Kincaid score is a validated readability metric with a goal reading level of 6 for middle school readability [72]. The Jargon score is the quantity of medical jargon used in the text, with a goal of as close to zero medical jargon as possible. We are continuing the fine-tuning process and testing additional questions against expert opinion before obtaining end-user feedback.

**Table 6:** Selected large language model performance. Notable improvements in scores toward goal values were observed.

Metrics	Before fine-tuning	After fine-tuning	Goal score
bleu@1	0.075	0.086	—^a^
bleu@2	0.044	0.047	—
bleu@3	0.027	0.028	—
bleu@4	0.018	0.017	—
meteor	0.135	0.117	—
rouge	0.106	0.089	—
Accuracy (1~5)	4.16	5	5
Readability (1~5)	4.63	4.98	5
Professional level (1~5)	4.58	4.98	5
Kincaid score^b^	8.54	7.17	6
Jargon score^c^	4.44	2.92	0

^a^Not applicable.

^b^A lower Kincaid score equates to an easier reading level.

^c^A lower Jargon scores equate to less medical jargon in text.

## Discussion

### Principal Findings

The purpose of this tutorial was to describe the iterative steps of developing a novel, intersectionality-based LLM to promote cardiovascular health among persons with HIV. This is one of the first studies to demonstrate the collaborative process between nursing and CS in personalized LLM development, as well as one of the first to specifically investigate AI integration for cardiovascular health promotion and education for people living with HIV. Other studies have investigated AI for cardiovascular and HIV screening, prevention, adherence, and risk prediction [7,33,34]. In addition, AI has been used for treatment plans, discharge summaries, and medical chatbots [7,35,36,46,47,77-79]. However, this work is unique in its intersectional lens, interdisciplinary methods, and varied opportunities for interventional application.

### Lessons Learned

There are a few key takeaways from developing this LLM, collaborating between disciplines, and considering potential applications. First, many resources need to be compiled to have enough tokens for LLM training. Second, gathering experts is vital for developing clinical judgment and managing patient safety. Third, fine-tuning of LLMs should include intersectional considerations, readability, and refusal prompts. Finally, this project highlights the benefit of interdisciplinary collaboration for health care innovation. However, developing communication strategies and shared learning opportunities may be potential methods to facilitate this collaboration. The initial challenges of this process came from inadequate premade LLM performance, high reading levels of sample sources, and difficulties with image and table scraping on websites. However, these challenges were mitigated by integrating expert feedback and fine-tuning for accessibility.

### Current State and Next Steps

Ongoing fine-tuning is in process to improve the readability and accuracy of our LLM. The LLM will be ready for end-user testing once the readability score consistently produces a fifth-grade level, the expert result rubric shows model-expert consensus, and the bias and screening challenges are successfully managed. After expert testing, end-user testing will evaluate the model’s usability, clarity, and relevance in real-world contexts. This phase will involve patients interacting with the model to pose questions and assess the helpfulness of its responses. Participants will provide structured, quantitative feedback on response quality, trustworthiness, and practical value, as well as identify any gaps or misunderstandings via open-ended, qualitative feedback. This input will be used to guide further model refinement, ensuring that the LLM not only aligns with expert standards but also meets the informational needs and expectations of its intended users. The rationale behind multiple iterations of expert feedback before end-user testing is to limit ethical concerns regarding inaccurate or biased information and the risk of potential patient harm. While bias-free writing references were included in the materials used to train this model [80], ongoing bias and information security testing are needed.

### Limitations and Strengths

This tutorial outlines our team’s development of an LLM aimed at promoting cardiovascular health among individuals living with HIV. Although the model demonstrates promising performance improvements, several limitations inherent to AI development and health care applications remain. First, the risk of algorithmic bias persists despite comprehensive mitigation strategies. Our approach combined multiple LLM architectures, external expert input, and integration of diverse, reputable data sources, including guidelines from the American Heart Association and insights from patient forums, to build a model that aspires to be broadly representative. Nevertheless, fully capturing the complexity of all user perspectives is challenging, and some residual bias may remain. Ongoing evaluation and refinement are required to further minimize any unintended biases. Second, excessive energy consumption is a notable concern in AI model development. We addressed this by using secure, energy-efficient servers and designing a lightweight architectural solution to reduce resource usage. While these measures contribute to more sustainable operation, wider energy considerations linked to large-scale model deployment remain an area for continued optimization. Third, ensuring the generalizability of the model across a wide range of populations is a key challenge. Although our development process incorporated diverse data sources and expert feedback to enhance the model’s applicability for all users, further validation across additional real-world settings and demographic groups is necessary to ensure that the model performs reliably in various contexts. Fourth, our custom evaluation metrics introduce both advantages and limitations. Building a domain-specific LLM, tailored to cardiovascular health in HIV care, allowed us to outperform general-purpose models on specialized tasks. However, our Accuracy, Readability, and Professionalism scores remain bespoke and carry inherent subjectivity. Experts from nursing, public health, and CS applied the rubric independently and reached consistent judgments, but some subjectivity persists. To anchor our new Readability and Professionalism scales to established tools, we conducted an analysis comparing them against Flesch–Kincaid readability levels and a third-party bias-detection measure, which showed encouraging alignment. Nevertheless, these bespoke metrics are not yet standardized and will need broader revalidation in patient comprehension studies before they can be adopted in other medical domains.

Despite these limitations, our work exhibits significant strengths. First, our weekly collaborative meetings among nursing, public health, and CS experts, including external consultants, ensured clinical rigor, technical soundness, and ongoing bias audits. Second, we structured data collection, prompt design, and QA pairs around the American Heart Association’s Life’s Essential 8 framework, guaranteeing alignment with current, peer-reviewed cardiovascular guidelines. Third, an intersectional and minority-stress lens guided our training data and prompts, reducing stigmatizing language and enhancing cultural relevance for overlapping marginalized identities. Fourth, we developed custom Accuracy, Readability, and Professionalism metrics tailored to patient-education goals, driving outputs toward sixth-grade reading levels and bias-free language, rather than relying on generic natural language processing benchmarks. Fifth, our iterative 4-step fine-tuning pipeline (GPT-4 QA generation, expert curation, low-rank adaptation tuning, and reinforcement learning from human feedback), combined quantitative rewards with qualitative expert review to progressively improve model performance. Sixth, by choosing a lightweight Llama-3.1-8B base model and energy-efficient servers, we minimized environmental impact without sacrificing output quality. Finally, our transparent documentation of resource scraping, benchmarking, and metric development provided a clear blueprint for replication or adaptation in other disease domains. These improvements provide a robust foundation for future efforts to refine the model further and extend its application in managing comorbid conditions across varying clinical populations.

### Implications for AI Research and Clinical Care

The CARDIO LLM has significant implications for both nursing clinical practice, public health policy, and research. In clinical practice, integration of the LLM into electronic health records could enhance patient discharge education. In addition, our LLM could be adapted for audiovisual platforms to support educational games, virtual or augmented reality scenarios, and telehealth services. Furthermore, there are multiple policy implications for health-related LLM development. These implications include warranting regulations for privacy, implementation in the clinical and community-based settings, ethical use, and consistent validation practices that are grounded in clinical evidence-based guidelines to ensure the safety and reliability of patient-facing AI tools. For research, the technology offers a platform for exploring personalized patient teaching through features like customizable avatars and integration of patient health data, as well as for developing advanced capabilities, such as patient reminders and resource connections. These innovations promise to expand the role of technology in enhancing patient engagement and advancing health care delivery.

### Conclusions

The purpose of this tutorial was to describe the development of an intersectionality-based LLM designed to promote cardiovascular health among individuals living with HIV. We found that a fine-tuned, Llama-based model shows promise in delivering personalized, provider-driven, culturally sensitive discharge education based on the American Heart Association’s “Life’s Essentials 8.” Integrating AI into practice requires careful consideration of ethical concerns, data quality, and ongoing expert evaluation to ensure accuracy, reliability, and patient safety. The collaboration between researchers, nursing, and computer scientists highlights the importance of interdisciplinary efforts in creating innovative and effective health care solutions. As we continue to refine and test this model, we aim to create a scalable, sustainable tool that supports equitable health promotion and advances public health efforts. Using a customized LLM to deliver health information can significantly improve patient understanding, support behavior changes, and advance health optimization.

## Appendix

Multimedia Appendix 1 Sample (not comprehensive) resource for large language model scraping.

Multimedia Appendix 2 Large language model scoring rubric for evaluators.

Multimedia Appendix 3 Sample question and answer pairs.

Multimedia Appendix 4 Substance use screening flowchart.

Multimedia Appendix 5 Sample multiturn conversations (with refusal and clarification options).

Multimedia Appendix 6 Group Relative Policy Optimization equation.

## Abbreviations

AI: artificial intelligence
BLEU: Bilingual Evaluation Understudy
CVD: cardiovascular disease
CDC: Centers for Disease Control and Prevention
WHO: World Health Organization
CS: computer science
HCP: health care professional
LLM: large language model
ML: machine learning
METEOR: Metric for Evaluation of Translation With Explicit Ordering
QA: question and answer.
ROUGE: Recall-Oriented Understudy for Gisting Evaluation
TRIPOD-LLM: Transparent Reporting of a Multivariable Prediction Model for Individual Prognosis or Diagnosis–Large Language Model

## Data Availability

The datasets generated or analyzed during this research are not publicly available due to ongoing model development, but are available from the corresponding author on reasonable request.

## References

[1] Aguirre A, Hilsabeck R, Smith T, Xie B, He D, Wang Z, Zou N (2024). Assessing the quality of chatGPT responses to dementia caregivers' questions: qualitative analysis. JMIR Aging.

[2] Alowais SA, Alghamdi SS, Alsuhebany N, Alqahtani T, Alshaya AI, Almohareb SN, Aldairem A, Alrashed M, Bin Saleh K, Badreldin HA, Al Yami MS, Al Harbi S, Albekairy AM (2023). Revolutionizing healthcare: the role of artificial intelligence in clinical practice. BMC Med Educ.

[3] Mandl KD (2025). How AI could reshape health care-rise in direct-to-consumer models. JAMA.

[4] Li Y, Li Y, Wei M, Li G (2024). Innovation and challenges of artificial intelligence technology in personalized healthcare. Sci Rep.

[5] Sarker I (2024). LLM potentiality and awareness: a position paper from the perspective of trustworthy and responsible AI modeling. Discov Artif Intell.

[6] ANA Center for Ethics and Human Rights (2022). The ethical use of artificial intelligence in nursing practice [Position statement]. American Nurses Association.

[7] Olaboye JA, Maha CC, Kolawole TO, Abdul S (2024). Artificial intelligence in monitoring HIV treatment adherence: A conceptual exploration. Int. J. Multidiscip. Res. Updates.

[8] Weiner EB, Dankwa-Mullan I, Nelson WA, Hassanpour S (2025). Ethical challenges and evolving strategies in the integration of artificial intelligence into clinical practice. PLOS Digit Health.

[9] Saad L (2025). Americans' Ratings of U.S. Professions Stay Historically Low. Gallup.

[10] Ronquillo CE, Peltonen L, Pruinelli L, Chu CH, Bakken S, Beduschi A, Cato K, Hardiker N, Junger A, Michalowski M, Nyrup R, Rahimi S, Reed DN, Salakoski T, Salanterä Sanna, Walton N, Weber P, Wiegand T, Topaz M (2021). Artificial intelligence in nursing: Priorities and opportunities from an international invitational think-tank of the nursing and artificial intelligence leadership collaborative. J Adv Nurs.

[11] Yakusheva O, Bouvier MJ, Hagopian COP (2025). How artificial intelligence is altering the nursing workforce. Nurs Outlook.

[12] Benavidez GA, Zahnd WE, Hung P, Eberth JM (2024). Chronic disease prevalence in the US: sociodemographic and geographic variations by zip code tabulation area. Prev Chronic Dis.

[13] Hacker K (2024). The burden of chronic disease. Mayo Clin Proc Innov Qual Outcomes.

[14] Ramos SR, Kang B, Jeon S, Fraser M, Kershaw T, Boutjdir M (2024). Chronic illness perceptions and cardiovascular disease risk behaviors in black and latinx sexual minority men with HIV: a cross-sectional analysis. Nurs Rep.

[15] Durstenfeld MS, Hill CL, Clare RM, Chiswell K, Sanders G, Gray S, Vicini J, Marsolo K, Okeke NL, Meissner EG, Thomas KL, Morse CG, Bloomfield GS, Pettit AC, Longenecker CT (2025). Association of cardiologist clinic visits with cardiovascular primary prevention outcomes among people with HIV from underrepresented racial and ethnic groups in the southern United States. J Am Heart Assoc.

[16] (2024). Addressing social determinants of health and chronic diseases. U.S. Centers for Disease Control and Prevention.

[17] Chin LL, Kershaw T, Hernandez-Ramirez RU, Ramos SR (2025). Racism-related stress, health outcomes, substance use, and PrEP attitudes among Asian sexual minority men. Sci Rep.

[18] Ramos SR, Lardier DT, Opara I, Turpin RE, Boyd DT, Gutierrez JI, Williams CN, Nelson LE, Kershaw T (2021). Intersectional effects of sexual orientation concealment, internalized homophobia, and gender expression on sexual identity and HIV risk among sexual minority men of color: a path analysis. J Assoc Nurses AIDS Care.

[19] Ramos SR, O'Hare OM, Hernandez Colon A, Kaplan Jacobs S, Campbell B, Kershaw T, Vorderstrasse A, Reynolds HR (2021). Purely behavioral: a scoping review of nonpharmacological behavioral and lifestyle interventions to prevent cardiovascular disease in persons living with HIV. J Assoc Nurses AIDS Care.

[20] Martin SS, Aday AW, Allen NB, Almarzooq ZI, Anderson CAM, Arora P, Avery CL, Baker-Smith CM, Bansal N, Beaton AZ, Commodore-Mensah Y, Currie ME, Elkind MSV, Fan W, Generoso G, et al (2025). 2025 Heart disease and stroke statistics: a report of US and global data from the American heart association. Circulation.

[21] Caceres BA, Streed CG, Corliss HL, Lloyd-Jones DM, Matthews PA, Mukherjee M, Poteat T, Rosendale N, Ross LM, American Heart Association Council on CardiovascularStroke Nursing; Council on Hypertension; Council on LifestyleCardiometabolic Health; Council on Peripheral Vascular Disease;Stroke Council (2020). Assessing and addressing cardiovascular health in LGBTQ adults: a scientific statement from the American heart association. Circulation.

[22] Streed CG, Beach LB, Caceres BA, Dowshen NL, Moreau KL, Mukherjee M, Poteat T, Radix A, Reisner SL, Singh V, American Heart Association Council on Peripheral Vascular Disease; Council on Arteriosclerosis‚ ThrombosisVascular Biology; Council on CardiovascularStroke Nursing; Council on Cardiovascular RadiologyIntervention; Council on Hypertension;Stroke Council (2021). Assessing and addressing cardiovascular health in people who are transgender and gender diverse: a scientific statement from the American heart association. Circulation.

[23] Scott J, Agarwala A, Baker-Smith CM, Feinstein MJ, Jakubowski K, Kaar J, Parekh N, Patel KV, Stephens J, American Heart Association Prevention Science Committee of the Council on EpidemiologyPreventionCouncil on CardiovascularStroke Nursing; Council on Lifelong Congenital Heart DiseaseHeart Health in the Young;Council on LifestyleCardiometabolic Health (2025). Cardiovascular health in the transition from adolescence to emerging adulthood: a scientific statement from the American heart association. J Am Heart Assoc.

[24] Kang B, Chin L, Camacho-Rivera M, Garza M, de Jesús Espinosa T, Cong X, Fraser M, Boutjdir M, Ramos SR (2025). Intervention mapping for systematic development of a community-engaged CVD prevention intervention in ethnic and racial sexual minority men with HIV. Front Public Health.

[25] Ramos SR, Reynolds H, Johnson C, Melkus G, Kershaw T, Thayer JF, Vorderstrasse A (2024). Perceptions of HIV-related comorbidities and usability of a virtual environment for cardiovascular disease prevention education in sexual minority men with HIV: formative phases of a pilot randomized controlled trial. J Med Internet Res.

[26] Ramos SR, Kang B, Jeon S, Fraser M, Kershaw T, Boutjdir M (2024). Chronic illness perceptions and cardiovascular disease risk behaviors in black and Latinx sexual minority men with HIV: a cross-sectional analysis. Nurs Rep.

[27] Hulbert L, Mensa-Wilmot Y, Rutledge S, Owens-Gary M, Skeete R, Cannon MJ (2025). Interests and preferences in programs to improve health among men with or at risk for type 2 diabetes in racial and ethnic minority groups, 2019. Prev Chronic Dis.

[28] Ghandakly E, Moudgil R, Holman K (2025). Cardiovascular disease in people living with HIV: risk assessment and management. Cleve Clin J Med.

[29] Fodeh S, Wang R, Murphy TE, Kidwai-Khan F, Leo-Summers LS, Tessier-Sherman B, Hsieh E, Womack JA (2024). BoneScore: a natural language processing algorithm to extract bone mineral density data from DXA scans. Health Informatics J.

[30] Womack JA, Murphy TE, Leo-Summers L, Bates J, Jarad S, Smith AC, Gill TM, Hsieh E, Rodriguez-Barradas MC, Tien PC, Yin MT, Brandt CA, Justice AC (2022). Predictive risk model for serious falls among older persons living with HIV. J Acquir Immune Defic Syndr.

[31] Nong P, Adler-Milstein J, Apathy NC, Holmgren AJ, Everson J (2025). Current use and evaluation of artificial intelligence and predictive models in US hospitals. Health Aff (Millwood).

[32] Khalifa M, Albadawy M (2024). Artificial intelligence for clinical prediction: Exploring key domains and essential functions. Comput Methods Programs Biomed Update.

[33] Eguavoen VO, Amadin FI, Nwelih E (2024). Cardiovascular disease risk prediction for people living with HIV using ensemble deep neural network.

[34] Marcus JL, Sewell WC, Balzer LB, Krakower DS (2020). Artificial intelligence and machine learning for HIV prevention: emerging approaches to ending the epidemic. Curr HIV/AIDS Rep.

[35] Fetrati H, Chan G, Orji R (2024). Chatbots for sexual health improvement: a systematic review. Int J Hum-Comput Interact.

[36] Birkun AA, Gautam A (2024). Large language model-based chatbot as a source of advice on first aid in heart attack. Curr Probl Cardiol.

[37] Ma Y, Achiche S, Tu G, Vicente S, Lessard D, Engler K, Lemire B, Laymouna M, de Pokomandy A, Cox J, Lebouché B, MARVIN chatbots Patient Expert Committee (2025). The first AI-based chatbot to promote HIV self-management: a mixed methods usability study. HIV Med.

[38] Singla A, Khanna R, Kaur M, Kelm K, Zaiane O, Rosenfelt CS, Bui TA, Rezaei N, Nicholas D, Reformat MZ, Majnemer A, Ogourtsova T, Bolduc F (2024). Developing a chatbot to support individuals with neurodevelopmental disorders: tutorial. J Med Internet Res.

[39] Hao Y, Holmes J, Waddle M, Yu N, Vickers K, Preston H, Margolin D, Löckenhoff C, Vashistha A, Ghassemi M, Kalantari S (2024). Outlining the borders for LLM applications in patient education: developing an expert-in-the-loop LLM-powered chatbot for prostate cancer patient education. ArXiv. Preprint posted online on September 27, 2024.

[40] Ramjee P, Sachdeva B, Golechha S, Kulkarni S, Fulari G, Murali K, Jain M (2025). CataractBot: An LLM-powered Expert-in-the-loop chatbot for cataract patients. Proc ACM Interact Mob Wearable Ubiquitous Technol.

[41] Kwok CS, Abramov D, Parwani P, Ghosh RK, Kittleson M, Ahmad FZ, Al Ayoubi F, Van Spall HGC, Mamas MA (2021). Cost of inpatient heart failure care and 30-day readmissions in the United States. Int J Cardiol.

[42] Luther B, Wilson R, Kranz C, Krahulec M (2019). Discharge processes: what evidence tells us is most effective. Orthop Nurs.

[43] Kang E, Tobiano GA, Chaboyer W, Gillespie BM (2020). Nurses' role in delivering discharge education to general surgical patients: a qualitative study. J Adv Nurs.

[44] Trivedi S, Corderman S, Berlinberg E, Schoenthaler A, Horwitz L (2023). Assessment of patient education delivered at time of hospital discharge. JAMA Intern Med.

[45] Horwitz LI, Moriarty JP, Chen C, Fogerty RL, Brewster UC, Kanade S, Ziaeian B, Jenq GY, Krumholz HM (2013). Quality of discharge practices and patient understanding at an academic medical center. JAMA Intern Med.

[46] Bass J, Bodimeade C, Choudhury N (2025). A quality improvement project of patient perception of AI-generated discharge summaries: a comparison with doctor-written summaries. Ann R Coll Surg Engl.

[47] Zaretsky J, Kim JM, Baskharoun S, Zhao Y, Austrian J, Aphinyanaphongs Y, Gupta R, Blecker SB, Feldman J (2024). Generative artificial intelligence to transform inpatient discharge summaries to patient-friendly language and format. JAMA Netw Open.

[48] Chandler RD, Warner S, Aidoo-Frimpong G, Wells J (2024). "What Did You Say, ChatGPT?" The use of AI in black women's HIV self-education: an inductive qualitative data analysis. J Assoc Nurses AIDS Care.

[49] Leidinger A, Rogers R, Das S, Green BP, Varshney K, Ganapini M, Renda A (2024). How are LLMs mitigating stereotyping harms? Learning from search engine studies. Proceedings of the Seventh AAAI/ACM Conference on AI, Ethics, and Society (AIES-24).

[50] Bauer GR, Lizotte DJ (2021). Artificial intelligence, intersectionality, and the future of public health. Am J Public Health.

[51] Ulnicane I (2024). Intersectionality in artificial intelligence: framing concerns and recommendations for action. Soc Incl.

[52] Hatem R, Simmons B, Thornton JE (2023). A call to address AI "Hallucinations" and how healthcare professionals can mitigate their risks. Cureus.

[53] Crenshaw K (1989). Demarginalizing the intersection of race and sex: a black feminist critique of antidiscrimination doctrine, feminist theory and antiracist politics. U Chi Legal F.

[54] Crenshaw K (1991). Mapping the margins: intersectionality, identity politics, and violence against women of color. Stanf Law Rev.

[55] Meyer IH (2003). Prejudice, social stress, and mental health in lesbian, gay, and bisexual populations: conceptual issues and research evidence. Psychol Bull.

[56] Frost DM, Meyer IH (2023). Minority stress theory: application, critique, and continued relevance. Curr Opin Psychol.

[57] Rivas‐Koehl M, Rivas‐Koehl D, McNeil Smith S (2023). The temporal intersectional minority stress model: reimagining minority stress theory. J Fam Theory Rev.

[58] Lloyd-Jones DM, Allen NB, Anderson CAM, Black T, Brewer LC, Foraker RE, Grandner MA, Lavretsky H, Perak AM, Sharma G, Rosamond W, American Heart Association (2022). Life's essential 8: updating and enhancing the American heart association's construct of cardiovascular health: a presidential advisory from the American heart association. Circulation.

[59] Shanableh A, Aderibigbe S, Omar M, Shabib A, Badran A, Baydoun E, Hillman JR (2022). Challenges and opportunities of multi-disciplinary, inter-disciplinary and trans-disciplinary research. Higher Education in the Arab World: Research and Development.

[60] (2025). Features comparison. PyMuPDF.

[61] Fenniak M (2025). pypdf 6.0.0. PyPI.

[62] (2025). Markitdown. GitHub.

[63] Ou J, Huang T, Zhao Y, Yu Z, Lu P, Ying R (2025). Experience retrieval-augmentation with electronic health records enables accurate discharge QA. ArXiv. Preprint posted online on May 28, 2025.

[64] Qiu W, Huang Z, Hu H, Feng A, Yan Y, Ying R (2025). MindLLM: a subject-agnostic and versatile model for fMRI-to-text decoding. ArXiv. Preprint posted online on Jun 6, 2025.

[65] Labrak Y, Bazoge A, Morin E, Gourraud P, Rouvier M, Dufour R (2024). Biomistral: a collection of open-source pretrained large language models for medical domains. ArXiv. Preprint posted online on Jul 17, 2024.

[66] Yang A, Yang B, Zhang B, Hui B, Zheng B, Yu B (2024). Qwen2. 5 technical report. ArXiv. Preprint posted online on Jan 3, 2025.

[67] Grattafiori A, Dubey A, Jauhri A, Pandey A, Kadian A, Al-Dahle A, Letman A, Mathur A, Schelten A, Vaughan A, Yang A (2024). The llama 3 herd of models. ArXiv. Preprint posted online on Nov 23, 2024.

[68] Achiam J, Adler S, Agarwal S, Ahmad L, Akkaya I, Aleman F, Almeida D, Altenschmidt J, Altman S, Anadkat S, Avila R (2024). Gpt-4 technical report. ArXiv. Preprint posted online on Mar 4, 2024.

[69] Hu E, Shen Y, Wallis P, Allen-Zhu Z, Li Y, Wang S, Wang L, Chen W (2021). Lora: Low-rank adaptation of large language models. ArXiv. Preprint posted online on Oct 16, 2021.

[70] Shao Z, Wang P, Zhu Q, Xu R, Song J, Bi X, Zhang H, Zhang M, Li Y, Wu Y, Guo D (2024). Deepseekmath: Pushing the limits of mathematical reasoning in open language models. ArXiv. Preprint posted online on Apr 27, 2024.

[71] Badarudeen S, Sabharwal S (2010). Assessing readability of patient education materials: current role in orthopaedics. Clin Orthop Relat Res.

[72] Walters KA, Hamrell MR (2008). Consent forms, lower reading levels, and using flesch-kincaid readability software. Drug Inf J.

[73] Taherdoost H, Madanchian M (2023). Artificial intelligence and sentiment analysis: a review in competitive research. Computers.

[74] Papineni K, Roukos S, Ward T, Zhu W (2002). Bleu: a method for automatic evaluation of machine translation.

[75] Banerjee S, Lavie A (2005). METEOR: an automatic metric for MT evaluation with improved correlation with human judgments. https://aclanthology.org/W05-0909.pdf.

[76] Lin CY (2004). Rouge: A package for automatic evaluation of summaries. InText Summarization Branches Out.

[77] Li M, Zhang H, Xia C, Zhang Y, Ji H, Shi Y, Duan L, Guo L, Liu J, Li X, Dong M, Chen J (2025). [Application practice of AI empowering post-discharge specialized disease management in postoperative rehabilitation of the lung cancer patients undergoing surgery]. Zhongguo Fei Ai Za Zhi.

[78] Santos M, Peyroteo M, Lapão L (2024). AI-powered post-discharge monitoring to prevent patients readmissions and reduce workforce burden. Eur J Public Health.

[79] Stanceski K, Zhong S, Zhang X, Khadra S, Tracy M, Koria L, Lo S, Naganathan V, Kim J, Dunn AG, Ayre J (2024). The quality and safety of using generative AI to produce patient-centred discharge instructions. NPJ Digit Med.

[80] Veldhuis CB, Cascalheira CJ, Delucio K, Budge SL, Matsuno E, Huynh K, Puckett JA, Balsam KF, Velez BL, Galupo MP (2024). Sexual orientation and gender diversity research manuscript writing guide. Psychol Sex Orientat Gend Divers.

