# Supplementary material for: Interdisciplinary Development and Fine-Tuning of CARDIO, a Large Language Model for Cardiovascular Health Education in HIV Care: Tutorial
Source: J Med Internet Res. 2025 Sep 12;27:e77053. doi: 10.2196/77053 (PMC12475882; doi:10.2196/77053)
Supplement: Multimedia Appendix 1 [file jmir_v27i1e77053_app1.docx]

Appendix 1

**Table 1:** Resource Selection for LLM Scraping

| **Domain** | **Website Link** |
| --- | --- |
| Cardiovascular General | <https://www.heart.org/en/healthy-living> |
| Cardiovascular General | <https://www.heart.org/en/about-us/heart-attack-and-stroke-symptoms> |
| Cardiovascular General | <https://www.heart.org/en/health-topics> |
| Cardiovascular General | <https://www.heart.org/en/healthy-living/healthy-lifestyle/lifes-essential-8> |
| Cardiovascular General | <https://professional.heart.org/en/guidelines-statements> |
| Screenings/ Prevention | <https://www.uspreventiveservicestaskforce.org/uspstf/recommendation-topics/information-for-consumers> |
| Screenings/Prevention | <https://www.sbm.org/healthy-living> |
| Cardiovascular General | <https://www.nhlbi.nih.gov/health> |
| Cardiovascular General | <https://www.nhlbi.nih.gov/es/salud> |
| Cardiovascular General | <https://www.nhlbi.nih.gov/resources?f%5B0%5D=language%3AEnglish&f%5B1%5D=language%3ASpanish> |
| DM2 | <https://www.niddk.nih.gov/health-information> |
| DM2 | <https://www.niddk.nih.gov/health-information/informacion-de-la-salud> |
| DM2 | <https://www.cdc.gov/diabetes/risk-factors/diabetes-risk-lgbtq.html> |
| DM2 | <https://www.lgbtqiahealtheducation.org/wp-content/uploads/2019/07/TFIE-35_LGBT-Diabetes-Brief_final2_pages.pdf> |
| Cardiovascular General | <https://www.ahajournals.org/doi/10.1161/CIR.0000000000001209> |
| Cardiovascular General | <https://www.ahajournals.org/doi/full/10.1161/CIR.0000000000001003> |
| SOGI General | <https://psycnet.apa.org/fulltext/2024-79040-001.html> |
| SOGI General | <https://translegislation.com/> |
| SOGI General | <https://www.aclu.org/legislative-attacks-on-lgbtq-rights-2024> |
| SOGI General | <https://medlineplus.gov/lgbtqiahealth.html> |
| SOGI General | <https://lgbtqhealthcaredirectory.org/> |
| SOGI General | <https://www.lgbtqiahealtheducation.org/resources/> |
| SOGI General | <https://www.hrc.org/resources> |
| Mental Health | <https://www.samhsa.gov/find-help> |
| Mental Health | <https://www.apa.org/topics> |
| Mental Health | <https://www.nimh.nih.gov/health> |
| Smoking | <https://smokefree.gov/> |
| Cholesterol | <https://www.ahajournals.org/doi/10.1161/CIR.0000000000000625> |
| HIV Care | <https://clinicalinfo.hiv.gov/en/guidelines> |
| HIV Care | <https://www.hiv.gov/hiv-basics> |
| HIV Care | <https://www.hivguidelines.org/> |
| HIV Care | <https://www.cdc.gov/hiv/index.html> |
| HIV Care | <https://www.cdc.gov/hivpartners/php/index.html> |
| HIV Care | <https://www.who.int/news-room/fact-sheets/detail/hiv-aids> |
| Sleep | <https://www.nhlbi.nih.gov/health/heart-healthy-living/sleep> |
| Medications | <https://www.drugs.com/> |
| General Health | <https://www.ncbi.nlm.nih.gov/books/NBK430685/> |
| DM2 | <https://pro.aace.com/clinical-guidance/diabetes> |
| DM2 | <https://professional.diabetes.org/standards-of-care> |
| Smoking | <https://goldcopd.org/2024-gold-report/> |
| Screenings/ Prevention | <https://www.cdc.gov/vaccines/hcp/imz-schedules/index.html> |
| Screenings/ Prevention | [https://www.cancer.org/cancer/types/colon-rectal-cancer/detection-diagnosis-staging/acs-recommendations.html](https://nam12.safelinks.protection.outlook.com/?url=https%3A%2F%2Fwww.cancer.org%2Fcancer%2Ftypes%2Fcolon-rectal-cancer%2Fdetection-diagnosis-staging%2Facs-recommendations.html&data=05%7C02%7Cryan.rullo%40yale.edu%7Cb7e656de07cf43cf95c608dd3b048a9f%7Cdd8cbebb21394df8b4114e3e87abeb5c%7C0%7C0%7C638731612894604873%7CUnknown%7CTWFpbGZsb3d8eyJFbXB0eU1hcGkiOnRydWUsIlYiOiIwLjAuMDAwMCIsIlAiOiJXaW4zMiIsIkFOIjoiTWFpbCIsIldUIjoyfQ%3D%3D%7C0%7C%7C%7C&sdata=oiOFVRHvCRST2gHn1X5g%2FwwfsB1EFobo3TKY1Q08tUM%3D&reserved=0) |
| Screenings/ Prevention | <https://www.asccp.org/clinical-practice/guidelines> |
| Screenings/ Prevention | [https://www.acog.org/clinical/clinical-guidance/practice-bulletin/articles/2017/07/breast-cancer-risk-assessment-and-screening-in-average-risk-women](https://nam12.safelinks.protection.outlook.com/?url=https%3A%2F%2Fwww.acog.org%2Fclinical%2Fclinical-guidance%2Fpractice-bulletin%2Farticles%2F2017%2F07%2Fbreast-cancer-risk-assessment-and-screening-in-average-risk-women&data=05%7C02%7Cryan.rullo%40yale.edu%7Cb7e656de07cf43cf95c608dd3b048a9f%7Cdd8cbebb21394df8b4114e3e87abeb5c%7C0%7C0%7C638731612894621155%7CUnknown%7CTWFpbGZsb3d8eyJFbXB0eU1hcGkiOnRydWUsIlYiOiIwLjAuMDAwMCIsIlAiOiJXaW4zMiIsIkFOIjoiTWFpbCIsIldUIjoyfQ%3D%3D%7C0%7C%7C%7C&sdata=doRfzTva17YkJWVjylOUjTdVPpnapn91kk7BjZDnzzg%3D&reserved=0) |
| Screenings/ Prevention | [https://www.asam.org/quality-care/clinical-guidelines/alcohol-withdrawal-management-guideline](https://nam12.safelinks.protection.outlook.com/?url=https%3A%2F%2Fwww.asam.org%2Fquality-care%2Fclinical-guidelines%2Falcohol-withdrawal-management-guideline&data=05%7C02%7Cryan.rullo%40yale.edu%7Cb7e656de07cf43cf95c608dd3b048a9f%7Cdd8cbebb21394df8b4114e3e87abeb5c%7C0%7C0%7C638731612894637743%7CUnknown%7CTWFpbGZsb3d8eyJFbXB0eU1hcGkiOnRydWUsIlYiOiIwLjAuMDAwMCIsIlAiOiJXaW4zMiIsIkFOIjoiTWFpbCIsIldUIjoyfQ%3D%3D%7C0%7C%7C%7C&sdata=7OwosPAD8vMxBgrBphHsX1Fy2kJbaYLQBjUcB8jTR44%3D&reserved=0) |
| Screenings/ Prevention | [https://www.asam.org/asam-criteria/implementation-tools/criteria-intake-assessment-form](https://nam12.safelinks.protection.outlook.com/?url=https%3A%2F%2Fwww.asam.org%2Fasam-criteria%2Fimplementation-tools%2Fcriteria-intake-assessment-form&data=05%7C02%7Cryan.rullo%40yale.edu%7Cb7e656de07cf43cf95c608dd3b048a9f%7Cdd8cbebb21394df8b4114e3e87abeb5c%7C0%7C0%7C638731612894654022%7CUnknown%7CTWFpbGZsb3d8eyJFbXB0eU1hcGkiOnRydWUsIlYiOiIwLjAuMDAwMCIsIlAiOiJXaW4zMiIsIkFOIjoiTWFpbCIsIldUIjoyfQ%3D%3D%7C0%7C%7C%7C&sdata=nLNzGrDdc4vx%2B5h02gXuaxhk75D8RZhM5P44ODD%2FXCk%3D&reserved=0) |
| Flesch Kincaid Grade Level (English) | <https://readable.com/readability/flesch-reading-ease-flesch-kincaid-grade-level/> |
| Fernandez Huerta Readability Index (Spanish) | <https://www.spanishreadability.com/fernandez-huerta-readability-index> |
| Therapeutic Communication Techniques | <https://www.mccc.edu/nursing/documents/NRS225TherapeuticCommunications.pdf> |
| Harm Reduction Strategies | <https://facesandvoicesofrecovery.org/2019/10/18/key-harm-reduction-strategies/?gad_source=1&gclid=Cj0KCQiAwtu9BhC8ARIsAI9JHalLjPURunzZs7MgV2uHLZ9hO-2g2hM7zgU-qE8zaFFxY2FLkEnN5koaAlQ9EALw_wcB> |
| Forum for Queries | <https://www.reddit.com/r/hivaids/?share_id=aoMnalJwXk4SBcVZtxc40&utm_content=1&utm_medium=ios_app&utm_name=ioscss&utm_source=share&utm_term=4> |
| Forum for Queries | <https://forums.poz.com/index.php?PHPSESSID=qe2trb4da2kmn0dch13402q9u2&action=forum> |
| Forum for Queries | <https://h-i-v.net/forums> |
