# Supplementary material for: Interdisciplinary Development and Fine-Tuning of CARDIO, a Large Language Model for Cardiovascular Health Education in HIV Care: Tutorial
Source: J Med Internet Res. 2025 Sep 12;27:e77053. doi: 10.2196/77053 (PMC12475882; doi:10.2196/77053)
Supplement: Multimedia Appendix 3 [file jmir_v27i1e77053_app3.docx]

Appendix 3

**Table 1:** Sample Q&A Pairs

| **Sample Model Q&A (Pre-Readability Score, English Only)** | | |
| --- | --- | --- |
| **Domain** | **Question** | **Answer** |
| Smoking | How can I quit smoking? | Commit to a quit day, choose a method for quitting (cold turkey or weaning down), talk to your doctor about medications to help quit, make a plan for when you quit on how to resist temptations, remove triggers, and have replacement activities, and then quit on your quit day! |
| Diet | How many calories should I eat in a day? | How many calories you should eat in a day depends on your age, gender, weight, and physical activity level. The nutrition label provides percentages based on a diet of 2,000 calories a day, but you should discuss with your doctor if this is appropriate for you. |
| DM2 | What are the risk factors for diabetes? | There are some non-modifiable risk factors for diabetes such as family history, race (Black, Asian, Latinx, Hispanic, Native American, Pacific Islander), age (>45), and history of gestational diabetes. There are also modifiable risk factors you can manage such as weight, physical activity, blood pressure, and smoking. |
| CV General | What are the signs of a heart attack? | Signs of a heart attack include chest pain (which may radiate to the arm or the jaw), shortness of breath, sweating, nausea, and light-headedness. These symptoms may be vaguer for women and people with diabetes. |
| HTN | What is a good blood pressure? | Normal blood pressure is considered a systolic (upper number) below 120 and diastolic (lower number) less than 80, so <120/<80. |
| Cholesterol | What foods have cholesterol? | Foods that come from animals all have cholesterol (meat, poultry, dairy). Saturated and trans fats can also increase cholesterol, which can be found in many foods including tropical oils (palm oil, coconut oil). |
| SOGI General | What are the main political issues facing LGBT people today? | Current legislation regarding limitations on free speech such as drag bans, curriculum censorship in schools, forced outing in schools, and religious exceptions for discrimination laws are some current political issues LGBTQIA+ people face today. |
| Mental Health | How can I differentiate between stress and a more serious mental health condition? | Stress is a normal response to life events, but persistent anxiety that interferes with daily activities might require professional help. |
| Screenings & Prevention | Why are cholesterol and blood pressure screenings essential for people with HIV? | HIV and antiretroviral therapy (ART) can increase cholesterol and blood pressure, which raises the risk of cardiovascular disease. Regular screenings help monitor and manage these risks |
| HIV Care | Can HIV be cured? | There is no cure for HIV, but it can be managed effectively with ART, allowing people to live long, healthy lives. Research on a potential cure is ongoing. |
| Sleep | How does sleep apnea affect cardiovascular health? | Sleep apnea increases the risk of hypertension, heart failure, and arrhythmias due to repetitive oxygen deprivation during sleep. |
| Exercise | Can exercise improve insulin sensitivity in people with prediabetes? | Yes, regular physical activity improves insulin sensitivity, reducing the risk of developing type 2 diabetes in people with prediabetes. |
| Weight | How does obesity contribute to the development of high blood pressure? | Obesity leads to increased blood volume and cardiac output, causing higher arterial pressure. Additionally, excess adipose tissue can cause hormonal imbalances that affect blood pressure regulation. |
