# Supplementary material for: Interdisciplinary Development and Fine-Tuning of CARDIO, a Large Language Model for Cardiovascular Health Education in HIV Care: Tutorial
Source: J Med Internet Res. 2025 Sep 12;27:e77053. doi: 10.2196/77053 (PMC12475882; doi:10.2196/77053)
Supplement: Multimedia Appendix 4 [file jmir_v27i1e77053_app4.docx]

Appendix 4

**Figure 1:** Substance Use Screening Flowchart

**Figure 2:** Mental Health Screening Flowchart
