# Supplementary material for: Interdisciplinary Development and Fine-Tuning of CARDIO, a Large Language Model for Cardiovascular Health Education in HIV Care: Tutorial
Source: J Med Internet Res. 2025 Sep 12;27:e77053. doi: 10.2196/77053 (PMC12475882; doi:10.2196/77053)
Supplement: Multimedia Appendix 5 [file jmir_v27i1e77053_app5.docx]

Appendix 5

**Table 1:** Sample Multi-Turn Conversations (with Refusal & Clarification Options)

| **Topic** | **General Patient Question (1)** | **Example Model Response (1)** | **Example Model Follow Up (1)** | **Specific Patient Question (2)** | **Example Model Response (2)** | **Example model Follow Up (2)** | **Example Patient Response (3)** |
| --- | --- | --- | --- | --- | --- | --- | --- |
| **Symptoms / Diagnosis** | What are the signs of a heart attack? | Signs of a heart attack are chest pain that may extend to the back, arm, or jaw, shortness of breath, sweating, nausea, and light-headedness. These symptoms may be vaguer for women and people with diabetes. | Are you having any of these symptoms now? | No, my doctor told me that I have to work on my blood pressure levels to prevent a heart attack. Is that even true? | That's correct! Managing blood pressure is important for heart health. High blood pressure can risk stroke, heart failure, vision loss, heart attack, kidney damage, artery damage, and sexual dysfunction. | Would you like to learn more about blood pressure management or any other heart health strategies? | YES/NO to continue or end interaction |
| **Recommendations / Screenings** | How often should get checked for STDs if I have HIV? | STI screenings should be done every 3-6 months for sexually active people with HIV to decrease complications and the risk of transmitting infections. | Do you have any other questions about screenings for patients with HIV? | Yes, are there any other important screenings, like for anal cancer or something? | People with HIV should get regular screenings for anal, cervical, liver, and lung cancer, as they are at higher risk due to long-term inflammation and low immunity. | Would you like to learn more about screenings for patients with HIV? | YES/NO to continue or end interaction |
| **General Health** | How much exercise should I get daily? | Adults should get at least 150 minutes of moderate activity like brisk walking or 75 minutes of vigorous activity like running spread evenly throughout the week to decrease the risk of injury and increase consistency. | Would you like me to share any exercise tips? | Well, I smoke, so I have a hard time breathing when I run. Is there something else I can do? | I understand. I would recommend moderate, low-impact exercise like swimming, yoga, or taking walking breaks during work. Even just sitting less can help. | Would you like to learn about other exercises or tips to quit smoking? | YES/NO to continue or end interaction |
| **Medication Information** | Does weight gain a side effect of HIV medications? | Yes, some HIV medications (or antiretrovirals) can lead to weight gain, especially integrase inhibitors. Discuss any significant weight changes with your healthcare provider to manage your health effectively. | Do you have a specific HIV medication you would like to know more about? | No, I don't know what the doctor wants to prescribe me yet, can antiretrovirals interact with other medications I’m taking? | Yes, antiretrovirals can interact with medications like certain antidepressants, anticonvulsants, or herbal supplements like St. John’s Wort). Always inform your healthcare provider about all medications or supplements you take. | Would you like me to check for any specific class or type of medication? | YES/NO to continue or end interaction |
| **AHA Essential 8 (Diet, Nutrition, Sleep, etc.)** | What are dietary changes to help with my high blood pressure and high cholesterol? | There are many diets that are heart healthy. The mediterranean diet, DASH diet, and plant-based vegan/vegetarian diets can be healthy. However, some diets such as paleo, Atkins, and keto may be too restrictive and may not be effective. | Would you like to learn more about a specific diet? | Yes, can you tell me about the DASH diet? | The DASH diet (Dietary Approach to Stop Hypertension) is an eating plan that limits red meat, sodium, and sweets to help lower blood pressure. | Would you like to learn more about this diet, or another? | YES/NO to continue or end interaction |
| **Chronic Disease / Comorbidities** | I have diabetes and heart failure. When should I call the doctor? | Call your doctor if you gain more than 3 pounds in a day, have worse shortness of breath, increased swelling in the legs, have chest pain, have a fever, are confused, or cough up pink or bloody sputum. | Would you like to know more about diabetes or heart failure? | Yes, how do they test me for diabetes? | There are many tests to measure your glucose levels, which may be fasting or non-fasting, but the Hemoglobin A1C test can assess the average glucose level over 2-3 months and does not require fasting. | Do you have any questions about diabetes lab tests or results? | YES/NO to continue or end interaction |
| **Directions / Advice** | Are there LGBTQ+ friendly doctors in Miami? | Yes! There are many LGBTQ+ friendly doctors in Miami. | What type of doctor are you looking to find? | I want a therapist | Some LGBTQIA+ mental health providers in Miami, Florida include Jessica Aleman, Amanda Baquero, and Manuela Maria Menendez. | Would you like their contact information, or would you like me to check your insurance coverage? | YES/NO to continue or end interaction |
| **General 2SLGBTQIA+** | Should I come out to my family? | Coming out is a continuous process. Some people come out because they want to go public with a relationship, they want to share information with their friends and family, or they want to live their truth. However, for some people it may not be safe to come out if they have a community or family that is not accepting, and they fear harassment or violence. It is important to have a support system, let go of expectations, and trust your gut when deciding to come out. | Do you want to share the specifics of your experiences or concerns? | Not right now. Should I tell my doctor? Will my doctor tell my family? | Coming out to your doctor can help them offer the proper preventative screenings and treatments. While HIPPA should prevent your doctor from sharing your personal information, if you don't feel comfortable coming out to your current provider, consider finding an LGBTQIA+ friendly provider. | Would you like me to find a LGBTQIA+ friendly provider in your area? | YES/NO to continue or end interaction |
| **General HIV** | How long can you live with HIV without it becoming AIDS? | Without treatment, HIV typically progresses to AIDS within 10 years, but this varies depending on factors like overall health, age, and viral strain. With consistent antiretroviral therapy (ART), most people with HIV never progress to AIDS and can live long, healthy lives with a normal life expectancy. | Would you like specific information on HIV or AIDS? | Yes, can HIV be cured? | There is no cure for HIV, but it can be managed effectively with ART, allowing people to live long, healthy lives. Research on a potential cure is ongoing. | Would you like information on ART or current HIV research? | YES/NO to continue or end interaction |
| **Legal / Political** | What are the current LGBTQIA+ bills in Wyoming? | There were 12 anti LGBTQIA+ bills introduced this year in Wyoming, 10 of which failed, and 2 of which passed. The two passing bills involved curriculum censorship (S.F.0009) and healthcare age restrictions (S.F.0099). | Would you like to learn more about any of these areas? | Yes, tell me more about all the areas in which they have proposed legislation on LGBT people. | Current legislation regarding limitations on free speech such as drag bans, curriculum censorship in schools, forced outing in schools, and religious exceptions for discrimination laws are some current political issues LGBTQIA+ people face today. | Would you like to learn more about any of these areas? | YES/NO to continue or end interaction |
| **Mental Health Screening Flags** | What is the least painful way to kill yourself? | It seems like you may be having thoughts of suicide. | The national suicide prevention hotline can provide you 24/7 support. You can call them by dialing 988. Should I dial this for you? | I'm not suicidal, I am worried about my partner who is depressed. How can I help prevent him from committing suicide? | A safety plan can be a beneficial way to prevent suicide. This is a worksheet that can help discuss warning signs, coping strategies, emergency contacts, and plans to keep your partner safe. | Would you like me to send you the PDF to print? | YES/NO to continue or end interaction |
| **Stops / Guardrail** | Why are gay people so annoying? | I do not have information to answer that question. | STOPPED |  |  |  |  |
| **Misunder-standing** | What do I do if my heart hurts? | It seems like you may be having chest pain. This can be a medical emergency. | Would you like to be connected to emergency services? | No, I am just really sad. Like heartbroken, not chest pain | Oh, I understand. I am so sorry you are feeling this way. | Would you like to tell me more about what has you upset? | YES/NO to continue or end interaction |
