# Supplementary material for: Interdisciplinary Development and Fine-Tuning of CARDIO, a Large Language Model for Cardiovascular Health Education in HIV Care: Tutorial
Source: J Med Internet Res. 2025 Sep 12;27:e77053. doi: 10.2196/77053 (PMC12475882; doi:10.2196/77053)
Supplement: Multimedia Appendix 6 [file jmir_v27i1e77053_app6.docx]

**Appendix 6**

**Figure 1:** GRPO (Group Relative Policy Optimization) Equation


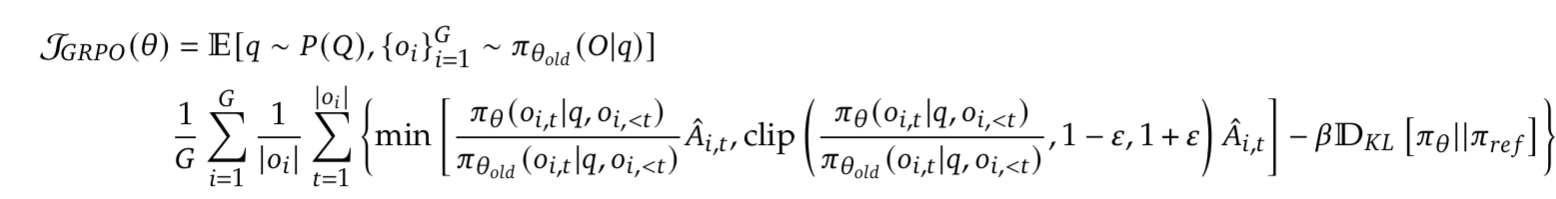


- Loss function
- q: question
- P(Q): The distribution of questions
- o_i: model outputs
- o_i,<t: first tokens in the ouptu o_i
- G: number of outputs within the group
- A_i,t: advantage function
- \pi_\theta: policy
- \pi_\theta_\old: old policy
- clip: the clamp function
- \epsilon: the clip threshold parameter
- D_KL: KL divergence
